# CT−based radiomics signature for differentiating pyelocaliceal upper urinary tract urothelial carcinoma from infiltrative renal cell carcinoma

**DOI:** 10.3389/fonc.2023.1244585

**Published:** 2024-01-18

**Authors:** Xiaoli Zhai, Penghui Sun, Xianbo Yu, Shuangkun Wang, Xue Li, Weiqian Sun, Xin Liu, Tian Tian, Bowen Zhang

**Affiliations:** ^1^ Department of Radiology, Beijing Chaoyang Hospital, Capital Medical University, Beijing, China; ^2^ CT Collaboration, Siemens Healthineers Ltd., Beijing, China; ^3^ Department of Pathology, Beijing Chaoyang Hospital, Capital Medical University, Beijing, China; ^4^ Huiying Medical Technology (Beijing) Co., Ltd., Beijing, China

**Keywords:** infiltrative renal cell cancer, pyelocaliceal upper urinary tract urothelial carcinoma, enhanced CT, differentiation, radiomics

## Abstract

**Objectives:**

To develop a CT-based radiomics model and a combined model for preoperatively discriminating infiltrative renal cell carcinoma (RCC) and pyelocaliceal upper urinary tract urothelial carcinoma (UTUC), which invades the renal parenchyma.

**Materials and methods:**

Eighty patients (37 pathologically proven infiltrative RCCs and 43 pathologically proven pyelocaliceal UTUCs) were retrospectively enrolled and randomly divided into a training set (n = 56) and a testing set (n = 24) at a ratio of 7:3. Traditional CT imaging characteristics in the portal venous phase were collected by two radiologists (SPH and ZXL, who have 4 and 30 years of experience in abdominal radiology, respectively). Patient demographics and traditional CT imaging characteristics were used to construct the clinical model. The radiomics score was calculated based on the radiomics features extracted from the portal venous CT images and the random forest (RF) algorithm to construct the radiomics model. The combined model was constructed using the radiomics score and significant clinical factors according to the multivariate logistic regression. The diagnostic efficacy of the models was evaluated using receiver operating characteristic (ROC) curve analysis and the area under the curve (AUC).

**Results:**

The RF score based on the eight validated features extracted from the portal venous CT images was used to build the radiomics model. Painless hematuria as an independent risk factor was used to build the clinical model. The combined model was constructed using the RF score and the selected clinical factor. Both the radiomics model and combined model showed higher efficacy in differentiating infiltrative RCC and pyelocaliceal UTUC in the training and testing cohorts with AUC values of 0.95 and 0.90, respectively, for the radiomics model and 0.99 and 0.90, respectively, for the combined model. The decision curves of the combined model as well as the radiomics model indicated an overall net benefit over the clinical model. Both the radiomics model and the combined model achieved a notable reduction in false-positive and false-negativerates, resulting in significantly higher accuracy compared to the visual assessments in both the training and testing cohorts.

**Conclusion:**

The radiomics model and combined model had the potential to accurately differentiate infiltrative RCC and pyelocaliceal UTUC, which invades the renal parenchyma, and provide a new potentially non-invasive method to guide surgery strategies.

## 1 Introduction

Urothelial cancers of the renal pelvis and collecting system constitute approximately 10%–15% of all renal tumors (1). Early pyelocaliceal upper urinary tract urothelial carcinoma (UTUC) is centered on the renal pelvis and calyces and grows in a centripetal direction. Most pyelocaliceal UTUCs can be diagnosed by their characteristic location. Open radical nephroureterectomy with bladder cuff excision is the standard treatment when pyelocaliceal UTUC invades the renal parenchyma (2). However, some infiltrative renal cell carcinomas (RCCs) can also grow into the renal sinus and invade the renal pelvis, which can mimic CT imaging manifestations of pyelocaliceal UTUC invading the renal parenchyma, making differential diagnosis challenging (3–5). In addition, radical nephrectomy is often applied for patients with infiltrative RCC (6), which is different from the surgical treatment of pyelocaliceal UTUC invading the renal parenchyma. Meanwhile, UTUC is prone to recurrence, and the patients mostly have a poor prognosis, requiring close clinical follow-up. Therefore, differential diagnosis of infiltrative RCC and pyelocaliceal UTUC preoperatively is essential.

Multiphasic multidetector-row CT (MDCT) scanning is the most common imaging modality for the detection and staging of UTUC and RCC (7), which can guide the subsequent strategy of imaging examination and treatment (8–11). Florian (12) demonstrated similar rates for detection, sensitivity, and specificity of metastases and local recurrence of RCC when comparing a dual-phase protocol with arterial and portal venous contrast to a single-phase protocol with portal venous contrast. Raza (13, 14) found that pyelocaliceal UTUC is more likely a solid, homogeneously enhancing mass centered on the collecting system and extended toward the ureteropelvic junction, with a focal pelvicalyceal filling defect and preserved renal outline. The accuracy of MDCT for the prediction of peritumoral invasion has positive and negative predictive values of 88.8% and 87.5%, respectively (14). Typically, RCCs appear as focal well-circumscribed masses and enhance avidly and heterogeneously with pseudocapsule (15, 16). In MRI, Wehrli found that pyelocaliceal UTUC exhibited a significantly lower normalized apparent diffusion coefficient (ADC) than RCC (17). In addition, Dursun found a higher SUV_max_ value in pyelocaliceal UTUC at 18−FDG PET/CT scanning (18). However, some infiltrative RCCs enhance poorly and homogeneously, with their imaging features overlapping with other cancers. Thus, more objective and quantitative parameters are required to identify infiltrative RCC and pyelocaliceal UTUC.

Radiomics is a quantitative analysis method based on medical images and uses a large number of algorithms to transform the region of interest (ROI) in medical images into high-dimensional features. It contains information on disease- and patient-specific processes that are imperceptible to the human eye (19, 20). It can be used to analyze the heterogeneity of an entire tumor based on hundreds of quantitative features and also quantitatively analyze the relationship between the biological and imaging characteristics of the tumor (21).

In this study, we aimed to assess the value of radiomics features and conduct a radiomics model and a combined model to differentiate pyelocaliceal UTUC that invades the renal parenchyma and infiltrative RCC based on enhanced CT images.

## 2 Methods

### 2.1 Patients

Data were collected through an electronic search of the picture archiving and communication system covering images recorded from January 2017 to December 2021. Two consecutive size-matched cohorts were established with the following inclusion criteria: 1) patients underwent nephrectomy, nephroureterectomy, or surgical resection of the renal lesions, and final diagnoses were based on histopathology, and 2) patients underwent preoperative four-phasic contrast-enhanced CT (CECT) scans. Exclusion criteria were prominent artifacts on CT images.

Finally, 80 patients (37 pathologically proven infiltrative RCCs and 43 pathologically proven pyelocaliceal UTUCs) were retrospectively enrolled and randomly divided into a training set (n = 56) and a testing set (n = 24) at a ratio of 7:3. Additionally, infiltrative RCCs were all clear cell carcinomas without rhabdoid or sarcomatoid differentiation, and pyelocaliceal UTUCs were all urothelial carcinomas.

### 2.2 CT technique

CECT images were obtained using three scanners: the SOMATOM Definition CT scanner, the SOMATOM Force CT scanner (Siemens Healthcare, Erlangen, Germany), and the Revolution Frontier CT scanners (General Electric Company, Chicago, IL, USA). Before scanning, the patients’ bodies were fixed, and they were instructed to remain still and breathe calmly. The scanning parameters were as follows: tube voltage of 120 kVp and automated tube current modulation and a variable setting of 280–300 mA. Other parameters were as follows: slice interval of 5 mm, slice thickness of 5 mm, and reconstructed section thickness of 1.25 mm.

Following unenhanced CT images, the arterial, portal, and delayed phase images were obtained in all patients. The portal phase that we used in this study was obtained with a delay of 70 seconds. All patients received non-ionic intravenous contrast material of approximately 60–80 mL. The contrast material was administered using mechanical power injectors. A contrast agent was injected into the anterior elbow vein or dorsal hand vein at a rate of 3 ml/s.

### 2.3 Clinical model development

Patient demographics and traditional CT imaging characteristics were used to construct the clinical model. Patient demographic characteristics were obtained from the picture archiving and communication system (PACS) of the hospital including gender, age, back pain, frequent urination, and painless hematuria. The traditional features based on the enhanced CT images were independently evaluated by two radiologists (SPH and ZXL, who have 4 and 30 years of experience in abdominal radiology, respectively). They were blinded to postoperative pathology. A consensus was reached through consultation in case of disagreement. The univariate logistic regression analysis was used to compare the differences in the patient demographics and traditional CT imaging characteristics between the infiltrative RCCs and pyelocaliceal UTUCs. The significant risk factors selected using the univariate logistic regression analysis were applied to the following multivariate logistic regression analysis to construct the clinical model.

### 2.4 Radiomics feature extraction and feature selection

**Figure 1:**
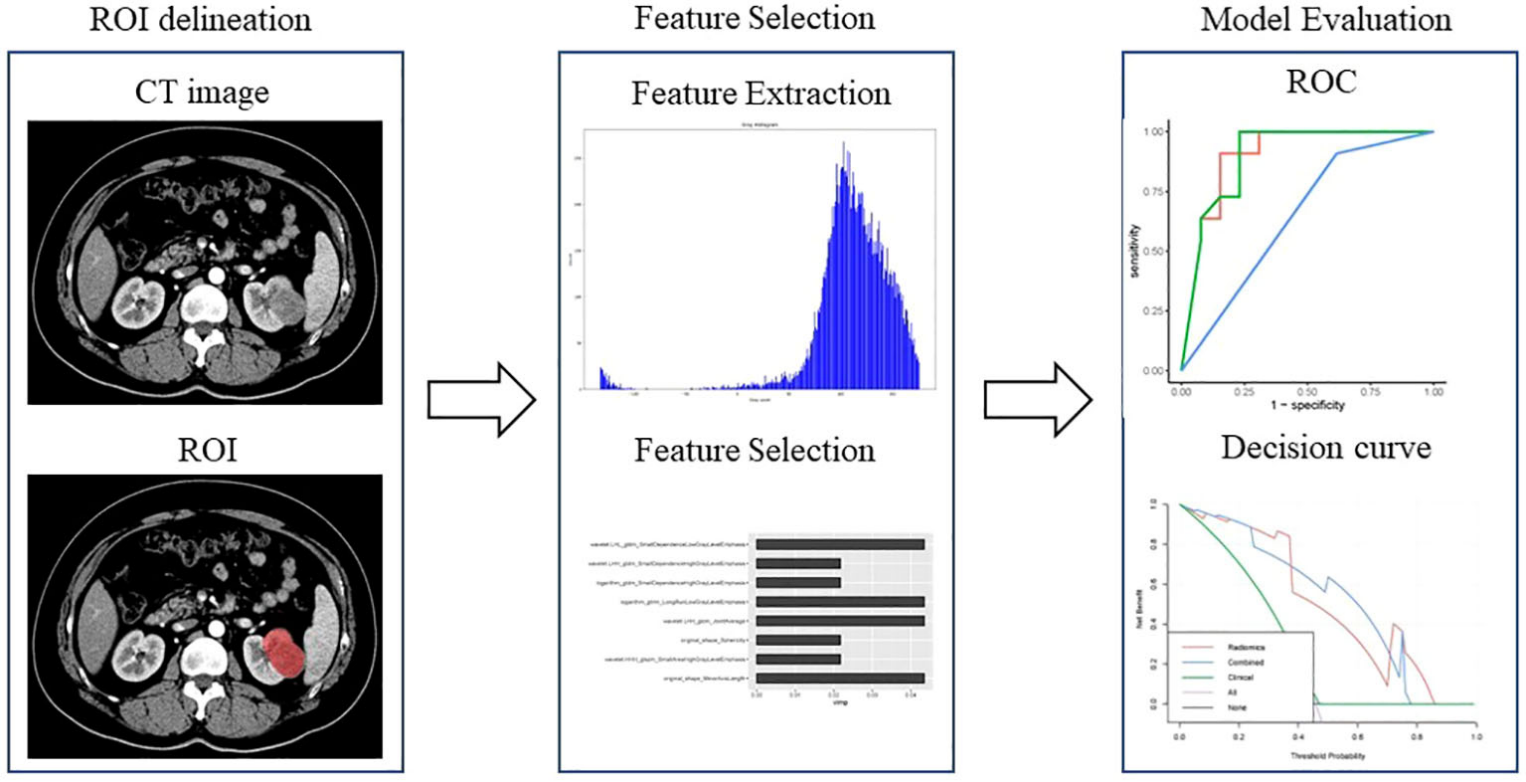
Workflow of the radiomics analysis to differentiate pyelocaliceal upper urinary tract urothelial carcinoma and infiltrative renal cell carcinoma.

**Figure 2:**
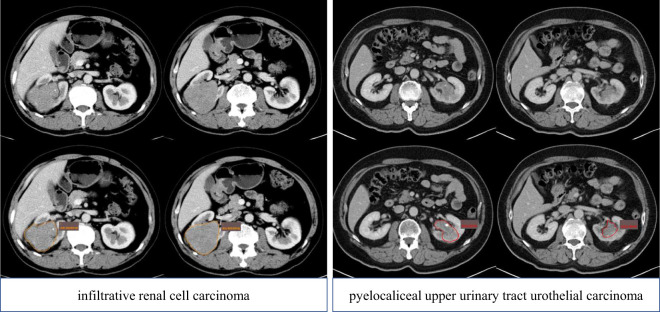
ROI delineation of pyelocaliceal upper urinary tract urothelial carcinoma and infiltrative renal cell carcinoma on the CT (portal phase). ROI, region of interest.

The workflow of the radiomics model construction was described in **Figure 1**. First, ROIs were manually drawn by junior radiologists using the ITK SNAP software (http://www.itksnap.org/pmwiki/pmwiki.php), and after that, ROIs were reviewed and approved by an expert radiologist (ZXL). An example of the manual segmentation is shown in **Figure 2**.

Then, the radiomics features were extracted based on the ROIs of the portal venous CT images. The radiomics features included intensity histogram features, shape and size features, and texture features such as gray-level co-occurrence matrix (GLCM), gray-level run length matrix (GLRLM), gray-level size zone matrix (GLSZM), neighborhood gray-tone difference matrix (NGTDM), and gray-level dependence matrix (GLDM). The definitions and names of the radiomics features were in accordance with the Image Biomarker Standardization Initiative (IBSI).

Although large numbers of features were extracted, not all features were beneficial to differentiating pyelocaliceal UTUC from infiltrative RCC. Therefore, the random forest-based Boruta algorithm was used to determine the features with the highest importance using the R package Boruta (22). Boruta is a random forest-based feature selection method. Boruta employs a recursive approach to disrupt the order of feature variables and assesses the importance of each feature to identify those with the highest relevance (22). Boruta is particularly advantageous for datasets with numerous predictor variables due to its superior computational efficiency (23).

### 2.5 Radiomics model construction

The radiomics model was built based on the features selected by Boruta using the R package randomForestSRC (24). Random forest consistently provides high prediction accuracy and is not prone to overfitting compared to other models. A 10-fold cross-validation on the model was applied to optimize the parameters of the random forest (RF) classifiers. The radiomics score (RF score) was calculated using a formula based on the radiomics features. RF score was used to build the radiomics model.

### 2.6 Combined model construction

The combined model was constructed by combining the significant factors of clinical factors and the RF score. The factors with *p* < 0.05 were considered significant predictors and used for developing the combined model.

### 2.7 Statistical analysis

Normal continuous variables are expressed as the mean ± standard deviation, whereas non-normal data are expressed as the median and interquartile range. Categorical variables are described as counts (percentages). Comparisons between groups were conducted using the t-test (normal data) or the Mann–Whitney U test (non-parametric data) for continuous variables and the chi-squared test for categorical variables.

The models were constructed using RF based on the feature sets with 10-fold cross-validation. In 10-fold cross-validation, the whole training set was randomly divided into 10 equal-sized subsets. A single subset was retained as the validation dataset, and the remaining four subsets were merged to create the training dataset. The cross-validation process was repeated 10 times, with each of the subsets used once as the validation dataset.

The receiver operating characteristic (ROC) curves and the area under the curve (AUC) value were used to assess the diagnostic efficacies of the three models. Delong’s test was performed to compare the AUCs of each model, and p-value < 0.0167 was considered statistically significant for multiple comparisons according to the Bonferroni correction. The net clinical benefits were assessed using decision curve analysis (DCA). R software (version 4.0.5 http://www.Rproject.org) was used for statistical analysis, and a two-sided *p* < 0.05 indicated statistical significance.

## 3 Results

### 3.1 Patients’ characteristics and the clinical model construction

A total of 80 patients (37 pathologically proven infiltrative RCCs and 43 pathologically proven pyelocaliceal UTUCs) were retrospectively enrolled and randomly divided into the training set (n = 56) and testing set (n = 24) at a ratio of 7:3. The patient demographics and traditional CT imaging characteristics between infiltrative RCCs and pyelocaliceal UTUCs are shown in **Table 1**. The univariate logistic regression analysis showed that painless hematuria, lesion volume, and intrapulmonary metastases were statistically significantly different between groups (*p* < 0.05). There was no statistically significant difference in hydronephrosis, stone, tumor calcification, and venous tumor thrombus between infiltrative RCCs and pyelocaliceal UTUCs (*p* > 0.05). After univariate logistic regression analysis, the features with statistically significant differences (*p* < 0.05) were applied to construct the multivariate logistic regression analysis. Multivariate analysis showed that painless hematuria was the risk factor for differentiating infiltrative RCCs and pyelocaliceal UTUCs (*p* < 0.05).

**Table 1:** Demographics and traditional CT features of the patients.

	RCC (*N* = 37)	UTUC (*N* = 43)	*p*
Male (%)	26 (70.27%)	27 (62.79%)	0.640
Age, year	63.38 [49.73–77.03]	67.98 [57.51–78.47]	0.100
Back pain (%)	17 (45.94%)	14 (32.56%)	0.320
Frequent urination (%)	3 (8.11%)	6 (13.95%)	0.494
Painless hematuria (%)	17 (45.94%)	39 (90.70%)	<0.001
Increased kidney volume (%)	27 (72.92%)	27 (62.79%)	0.174
Hydronephrosis (%)	10 (27.02%)	20 (46.51%)	0.118
Stone (%)	8 (21.62%)	13 (30.23%)	0.537
Tumor calcification (%)	3 (8.11%)	5 (11.63%)	0.719
Venous tumor thrombus (%)	12 (32.43%)	19 (44.19%)	0.398
Intrapulmonary metastases (%)	3 (8.11%)	12 (27.91%)	0.048
Left kidney (%)	21 (56.76%)	16 (37.24%)	0.072
Lesion volume	233 [73.50–346.00]	36.1 [25.5–178]	<0.001

RCC, renal cell carcinoma; UTUC, upper urinary tract urothelial carcinoma.

### 3.2 Radiomics model

**Figure 3:**
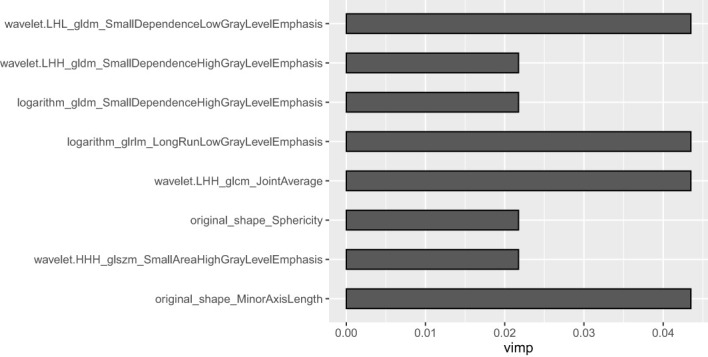
Weights of the eight radiomics features in the radiomics model.

A total of 1,688 radiomics features were extracted from the portal venous CT images. After the Boruta analysis, eight features were extracted to construct the final model (detailed information on the selected features is shown in **Figure 3**). Then, RF models were conducted, and the RF scores of the training set and the testing set were calculated. The RF models of the training set and the testing set performed well with AUC values of 0.95 (95%*CI*: 0.88–1.00) and 0.90 (95%*CI*: 0.77–1.00), respectively. In addition, the RF score was lower in the pyelocaliceal UTUC groups than in the infiltrative RCC groups, with *p* < 0.001.

### 3.3 Combined model

**Figure 4:**
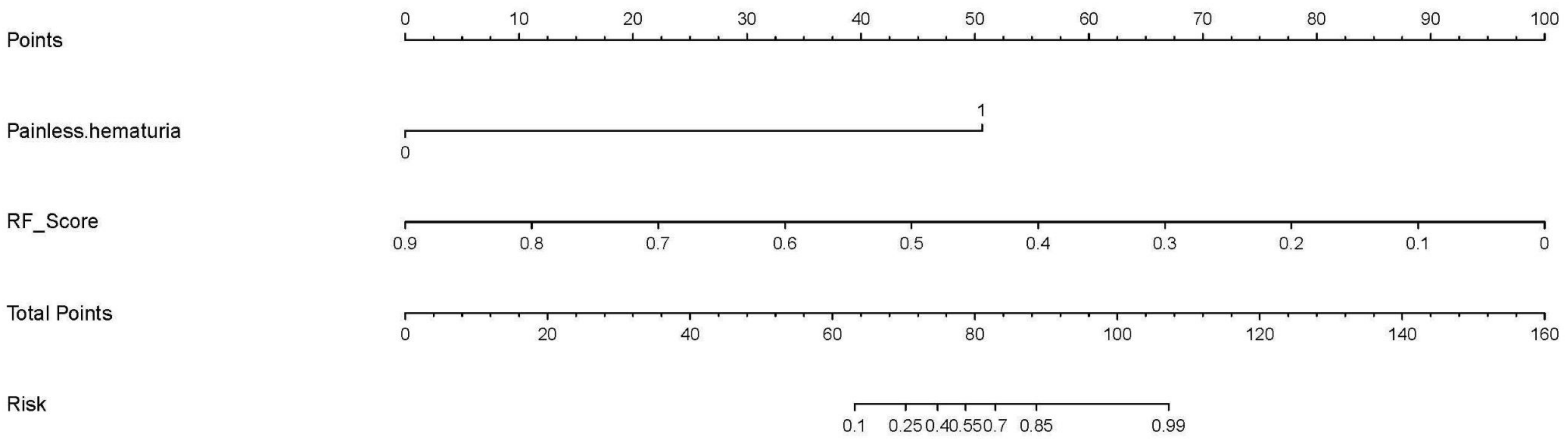
An individualized nomogram based on RF score and clinical features. RF, random forest.

**Figure 5:**
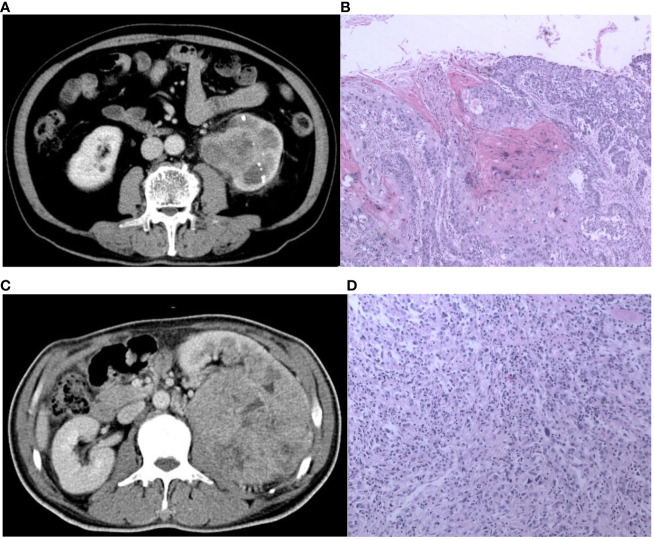
The CT and pathological images of the pyelocaliceal upper urinary tract urothelial carcinoma and infiltrative renal cell carcinoma. **(A)** A 60-year-old man with left kidney stones, hematuria, and left waist soreness. CT images show enlarged volume of the left kidney, multiple high-density nodular shadows in the parenchyma, and obvious expansion of the left renal pelvis with blurred edges, heterogeneous enhancement, and unclear boundary. **(B)** Pathological images (H&E, ×100) showed an invasive urothelial carcinoma infiltrating the muscle layer of the renal pelvis and infiltrating the renal parenchyma and finally diagnosed as pyelocaliceal upper urinary tract urothelial carcinoma, which was consistent with the judgment of nomogram. **(C)** A 56-year-old man with lower back pain and hematuria for more than 3 months. CT image showed a huge soft tissue mass in the left kidney, with heterogeneous enhancement, unclear boundaries, a low-density filling defect in the left renal vein, and an enlarged lymph node in the left retroperitoneum. **(D)** Pathological images (HE, 100×) showed a transparent cell carcinoma of grade 3 with necrosis, invading the renal capsule, renal pelvis mucosa, and renal sinus fat, and it was finally diagnosed as infiltrative RCC, which was not consistent with the judgment of nomogram.

The multivariate logistic regression analysis (as shown in **Table 2** and Appendix **Table 3**) indicated that painless hematuria and RF score are the independent risk factors, and a combined model was constructed based on the two factors. An individualized nomogram that incorporated the two predictive factors based on the combined model in the training cohort was constructed to differentiate infiltrative RCC and pyelocaliceal UTUC, which is shown in **Figure 4**. **Figure 5** shows a typically pyelocaliceal UTUC, which was consistent with the judgment of nomogram and an infiltrative RCC incorrectly identified as pyelocaliceal UTUC by nomogram.

**Table 2:** The univariate and multivariate logistic regression analyses of the patients.

	Univariate		Multivariate (clinical model)		Multivariate (combined model)	
	β	*p*	β	*p*	β	*p*
Male (%)	−0.278	0.640		0.65		
Age, year	0.0248	0.100				
Back pain (%)	−0.983	0.320				
Frequent urination (%)	0.932	0.494				
Painless hematuria (%)	2.780	<0.001	2.383	0.002	7.803	0.017
Increased kidney volume (%)	−1.820	0.174				
Hydronephrosis (%)	0.260	0.118				
Stone (%)	0.236	0.537				
Tumor calcification (%)	1.449	0.719				
Venous tumor thrombus (%)	0.260	0.398				
Intrapulmonary metastases (%)	1.609	0.048				
Left kidney (%)	−1.056	0.072				
Lesion volume	−0.002	0.037				
RF score	−9.091	<0.001			−17.127	0.005

RF, random forest.

**Table 3:** Pairwise comparisons of AUCs of the clinical model, radiomics model, and combined model.

	AUCs			*p* (0 *vs.* 1)		*p* (0 *vs.* 2)	*p* (1 *vs.* 2)
	Clinical models (0)	Radiomics models (1)	Combined models (2)				
Training cohort	0.61 (0.49–0.74)	0.95 (0.89–1.00)	0.99 (0.98–1.00)	<0.001		<0.001	0.104
Testing cohort	0.52 (0.33–0.71)	0.90 (0.77–1.00)	0.90 (0.77–1.00)	0.001		<0.001	0.927

AUC, area under the curve.

### 3.4 Comparison of the three models

**Figure 6:**
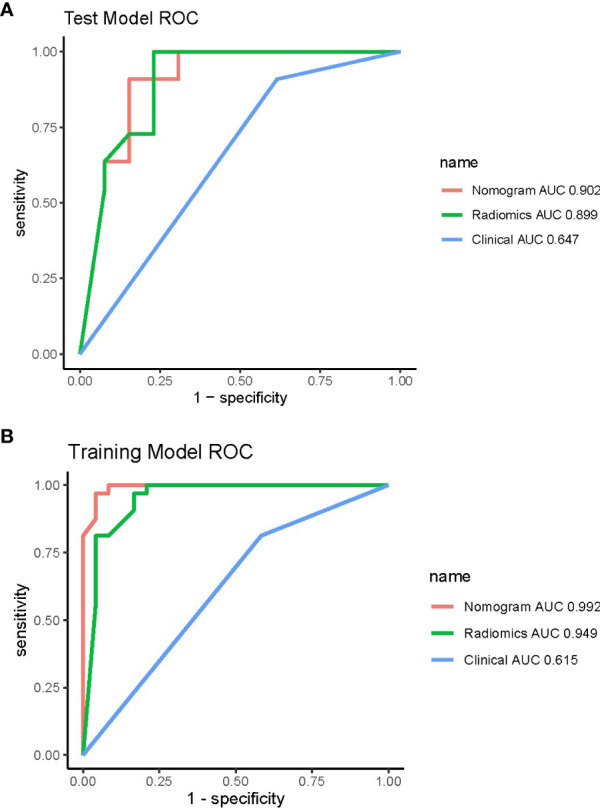
ROC curves of the three models in the training **(A)** and testing **(B)** cohorts. ROC, receiver operating characteristic.

**Figure 7:**
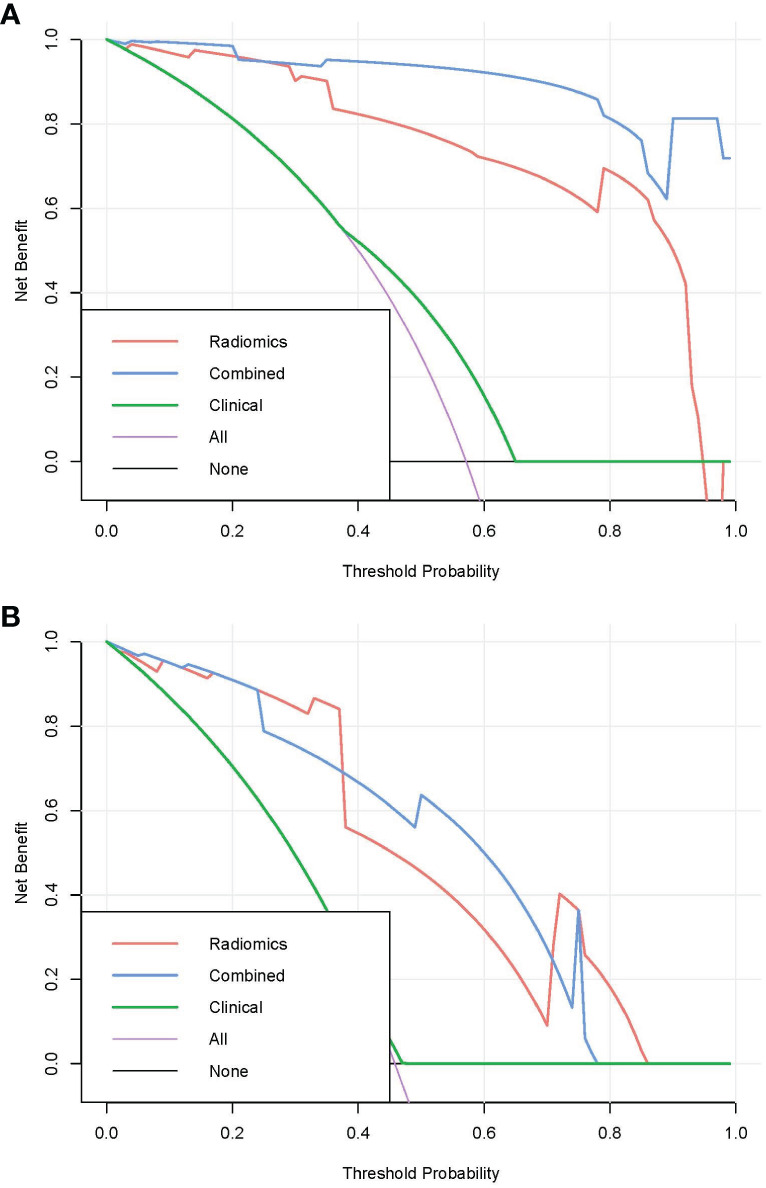
Decision curves for the three models in the training **(A)** and testing **(B)** cohorts.

Pairwise comparisons of the AUCs of the clinical model, radiomics model, and combined model were performed using Delong’s test. As **Table 3** shows, in the training cohort, the AUC of the combined model as well as the radiomics model was significantly higher than that of the clinical model (*p* < 0.001). In the testing cohort, the AUC of the combined model as well as the radiomics model was significantly higher than that of the clinical model (*p* < 0.001). In the training and testing sets, the AUC of the combined model was slightly higher than that of the radiomics model, although not statistically significant (all *p* > 0.0167). The ROC curves for the clinical model, radiomics model, and combined model are shown in **Figure 6**. The decision curves for the three models showed that the net clinical benefit for the combined model as well as the radiomics model was higher than that of the clinical model, which is shown in **Figure 7**.

### 3.5 Comparison of the radiomics model and the radiologists’ visual assessment

We included the confusion matrix and accuracy of the visual assessments conducted by two radiologists, as well as the machine learning models, in the attached appendix (**Tables 1**, **2**). The results demonstrated that both the radiomics model and the combined model achieved a notable reduction in false-positive and false-negativerates, resulting in significantly higher accuracy compared to the visual assessments in both the training and testing cohorts.

## 4 Discussion

Accurate preoperative determination of infiltrative RCC and pyelocaliceal UTUC plays a crucial role in treatment decisions and follow-up strategies. In this study, we first developed a pretreatment CT-based radiomics model and a combined model to distinguish infiltrative RCC and pyelocaliceal UTUC *via* contrast-enhanced CT with a satisfactory discriminatory performance. Our results indicated that the combined model performed best for distinguishing these two malignancies.

The univariate logistic regression analysis of traditional features showed that pyelocaliceal UTUC patients were more likely to have painless hematuria and intrapulmonary metastases, which is consistent with previous studies (25). The most classical symptom of pyelocaliceal UTUC is painless hematuria, and pyelocaliceal UTUC patients often exhibit symptoms earlier than RCC patients. Pyelocaliceal UTUCs invade and metastasize more easily because of the thin layer of surrounding ureteral adventitia containing an extensive plexus of blood vessels and lymphatic channels.

The multivariate logistic regression analysis showed that pyelocaliceal UTUC patients were more likely to have painless hematuria and lower RF scores. Pyelocaliceal UTUC patients have lower RF scores, which means that they have lower heterogeneous enhancement. Pyelocaliceal UTUCs have infiltrative hypo-vascular masses coexisting with a filling defect in the adjacent collecting system or amputation of a calix, renal shape preservation, the absence of cystic or necrotic change, homogeneity of the tumor, and extension into the ureteropelvic junction. However, RCCs are soft tissue attenuation and are sometimes accompanied by necrosis and calcification, which have stronger or irregular enhancement during the corticomedullary phase (26, 27).

Although radiomics analysis has already been applied for differentiating renal tumors, previously published studies mostly focused on identifying benign and malignant renal lesions or distinguishing different types of RCCs such as clear cell renal cell carcinoma, renal papillary cell carcinoma, and chromophobe cell renal cell carcinoma (28). We first used radiomics analysis to differentiate clear cell renal cell carcinoma and urothelial carcinomas with good accuracy. In addition, we also compared the accuracy of the combined model and that of two expert radiologists, which showed better performance of the combined model.

Several limitations of our study have to be considered. The study was a retrospective data analysis; therefore, the quality of CT imaging in some cases may not be so satisfactory, and bias produced among different CT machines was hard to control. Our study merely investigated the portal venous phase CT images to develop a radiomics signature. Radiomics features extracted from non-enhanced CT images as well as arterial and venous phase images could provide additional information for better discriminatory performance. In addition, image preprocessing such as resampling was not performed in this study, which may limit the reproducibility of our findings.

## 5 Conclusion

The RF radiomics model and combined model can improve the accuracy of differentiating pyelocaliceal upper urinary tract urothelial carcinoma, which invades the renal parenchyma from infiltrative renal cell carcinoma, and provide a new potentially non-invasive method to guide surgery strategies.

## Data availability statement

The original contributions presented in the study are included in the article/**Supplementary Material**. Further inquiries can be directed to the corresponding author.

## Ethics statement

Ethical approval was not required for the study involving humans in accordance with the local legislation and institutional requirements.

## Author contributions

XZ was responsible for clinical data collection and final ROI auditing. PS was responsible for drawing ROI, writing the article. SW was responsible for reviewing the article. XY and WS were responsible for radiomics analysis. XLi was responsible for pathological analysis. XLiu, TT and BZ were responsible for drawing ROI. All authors contributed to the article and approved the submitted version.
