# Supplementary material for: CT−based radiomics signature for differentiating pyelocaliceal upper urinary tract urothelial carcinoma from infiltrative renal cell carcinoma
Source: Front Oncol. 2024 Jan 18;13:1244585. doi: 10.3389/fonc.2023.1244585 (PMC10830825; doi:10.3389/fonc.2023.1244585)
Supplement: Supplementary file 1 [file DataSheet_1.pdf]

Appendix

Table.1 Confusion matrix and accuracy of visual assessment, clinical model, radiomics model and combined model in train cohort.

	TP	TN	FP	FN	Accuracy
Visual assessment from Radiologists 1	27	2	22	5	52(38-65)
Visual assessment from Radiologists 2	29	3	21	3	57(43-70)
Clinical Model	26	10	14	6	64 (54-75)
Radiomic Model	31	20	4	1	91 (84-98)
Combined Model	31	23	1	1	96 (91-100)

Table.2 Confusion matrix and accuracy of visual assessment, clinical model, radiomics model and combined model in test cohort.

	TP	TN	FP	FN	Accuracy
Visual assessment from Radiologists 1	8	2	11	3	42(22-63)
Visual assessment from Radiologists 2	8	1	12	3	38(19-59)
Clinical Model	8	4	9	3	50 (33-67)
Radiomic Model	11	9	4	0	83 (71-96)
Combined Model	11	9	4	0	83 (71-96)

Table 3. Adjust R-squared for Clinical model and Combined model.

	Adjust R-squared
Clinical model	0.244
Combined model	0.860
